# Metformin and butyrate attenuate chronic radiation proctitis by alleviating inflammation and macrophage senescence

**DOI:** 10.3389/fimmu.2026.1802803

**Published:** 2026-04-02

**Authors:** Mau-Shin Chi, Huan-I Jen, Shu-Yi Ho, Kai-Lin Yang, Hui-Ling Ko, Kwan-Hwa Chi

**Affiliations:** 1 Department of Radiation Therapy & Oncology, Shin Kong Wu Ho-Su Memorial Hospital, Taipei, Taiwan; 2 Institute of Veterinary Clinical Science, School of Veterinary Medicine, National Taiwan University, Taipei, Taiwan; 3 School of Medicine, Fu Jen Catholic University, New Taipei City, Taiwan

**Keywords:** butyrate, cellular senescence, chronic radiation proctitis, metformin, radiotherapy, senomorphic

## Abstract

**Introduction:**

Chronic radiation proctitis (RP) is characterized by persistent inflammation and impaired tissue repair. This study investigates the potential of a metformin–butyrate (MeBu) combination to modulate radiation-induced senescence-associated changes in macrophages to mitigate chronic injury.

**Materials and methods:**

BALB/c mice received a 15 Gy fraction of rectal brachytherapy. From weeks 4 to 8 post-irradiation, mice were treated with rectal enemas of metformin, butyrate, or the MeBu combination. Tissue histology (H&E and Masson’s Trichrome staining), macrophage polarization (iNOS/CD163) were evaluated. Effects on senescence markers were analyzed in irradiated bone marrow–derived macrophages (BMDMs) using SA-β-gal staining and qPCR for *p16* and *p21*. A composite Senescence Burden Index (SBI) was developed to integrate transcriptional senescence signals.

**Results:**

MeBu treatment was associated with a reduction in mucosal fibrosis and a phenotypic shift in macrophages toward a more reparative M2-like profile (increased CD163/iNOS ratio). In BMDMs, MeBu significantly reduced SA-β-Gal positivity and suppressed *p21* expression (p = 0.0074), with a downward trend in *p16* (p = 0.0568). The integrated SBI demonstrated that MeBu significantly attenuated the overall senescence burden compared to the irradiated group (*p* < 0.01). This combined effect was more robust than that of either metformin or butyrate alone.

**Conclusion:**

Our findings suggest that the MeBu combination may attenuate chronic RP by modulating macrophage-associated senescence and inflammation. These results indicate that metabolic-based senomorphic strategies hold potential to mitigate chronic inflammatory sequelae following pelvic radiotherapy.

## 1 Introduction

Chronic radiation proctitis (RP) is a significant complication of radiotherapy for pelvic malignancies, with an incidence of 5% to 21% that increases in correlation with higher radiation doses and larger irradiated volumes (1–3). Pathologically, acute RP is marked by inflammation and infiltration of immune cells into mucosa. Chronic RP often shows less active inflammation but includes vascular ectasia and fibrotic tissue changes. This condition is now referred as radiation-associated vascular ectasias (RAVE), which involves abnormal dilation and distortion of blood vessels, as well as fibrosis of the endothelial lining, resembling what is seen in gastric antral vascular ectasia (4, 5).

Fibrosis in RP is mainly driven by chronic inflammation that fails to resolve, with macrophages playing a central role (6). Macrophages are phenotypically plastic, which the M1 macrophages promote inflammation by releasing pro-inflammatory cytokines, while the M2 macrophages counteract this response by secreting anti-inflammatory factors and supporting tissue repair (7). During prolonged inflammation, macrophages often shift toward an M2-dominant phenotype, as in the senescence and chronic inflammatory conditions (8). The senescent macrophages lose their phagocytic capacity and instead amplify inflammation via the senescence-associated secretory phenotype (SASP) (9, 10). While irradiation drives vascular dysfunction in RAVE by inducing senescence in pericytes and endothelial cells, characterized by increased expression of SA-β-galactosidase (SA-β-Gal), *p16*, and *p21* (11, 12), current treatments often neglect this underlying immune-senescence (13, 14).

Metformin and butyrate have shown individual potential in metabolic and epigenetic modulation (15–18). These two agents work synergistically to promote anti-aging pathways and reduce systemic inflammation (19). A combined metformin-butyrate (MeBu) approach has shown potential in managing steroid-refractory chronic RP in a case report (20). This study aims to investigate how the MeBu combination targets these cellular aging processes to promote repair.

## 2 Materials and methods

### 2.1 Animal model and induction of RP

Eight-week-old female BALB/c mice (20–24 g; LASCO Biotech, Taipei, Taiwan) were used to provide a standardized adult immune baseline. Mice were randomly assigned to five groups (n = 9 per group), with data derived from three independent biological replicates (n = 3 per group per replicate) to ensure reproducibility. All animal work was approved by the institutional animal care and use committee (Protocol Number: 111SKH010). The animals were kept in propylene cages (4 mice/cage) with environmental enrichment and a temperature-controlled room (23 ± 1 °C) with 50-60% relative humidity. The mice were submitted to a 12h/12h light-dark cycle with free access to food (Land O’Lakes, Inc., US) and water.

Mice exposed to treatment were first anesthetized with acepromazine (0.75 mg/kg) intraperitoneally and then immobilized in the prone position. The rectum of the mice, approximately 2.9 mm in diameter, was divided into three segments: the rectal portion (0–10 mm), the intermediate portion (10–20 mm), and the colonic portion (20–30 mm), with anus marked as the zero coordinate (21, 22). After immobilization, a single-channel blind-ended lumen catheter with a 2 mm outer diameter was inserted into the rectum until its tip reached 10 mm from the anal verge. Each animal was irradiated individually using a high dose rate (HDR) brachytherapy after-loading system by microSelectron^®^ Digital (Elekta, Stockholm), with a radioactive Iridium 192 source delivered through the inserted lumen catheter. The animals were divided into irradiated and sham groups, with a single fraction of 5Gy, 10 Gy, 15 Gy, or 20 Gy prescribed at 5 mm off-axis to the rectal mucosa in the irradiated groups. The treatment plan was generated using the Oncentra^®^ Brachytherapy planning system (Elekta, Stockholm).

Mice were closely monitored for survival and euthanized upon reaching predefined humane endpoints, including signs of imminent death, markedly reduced mobility, inability to maintain an upright posture, ataxia, or abnormal respiration. Euthanasia was performed by inhalation of 5% isoflurane until complete cessation of respiration, followed by cervical dislocation to ensure death. Rectal tissues were immediately harvested postmortem, fixed in 10% neutral-buffered formalin for 24 hours, and subsequently embedded in paraffin. Tissue sections of 5 µm thickness were prepared for histopathological and immunohistochemical analyses. All animal experiments were conducted in accordance with institutional guidelines and approved by the Institutional Animal Care and Use Committee.

### 2.2 Therapeutic enema administration for RP

To evaluate the therapeutic efficacy of MeBu, mice with RP were administered enemas containing metformin (Sigma-Aldrich, St. Louis, MO, USA, Cat# 317240), sodium butyrate (Sigma-Aldrich, St. Louis, MO, USA, Cat# 303210), or combination (MeBu). Each agent was prepared in sterile saline at a concentration of 10% and delivered in a volume of 50\muL, corresponding to a dose of 200mg/kg for a standard 25 gm mouse. This dose and volume were selected to ensure adequate mucosal coverage and experimental reproducibility while minimizing systemic toxicity. Beginning at week 4 post-irradiation, animals were randomly assigned to receive daily rectal enemas for four weeks. Treatments were administered via a flexible catheter inserted into the distal rectum to ensure accurate delivery of the defined volume. At week 8, mice were euthanized, and rectal tissues were harvested for histopathological analysis.

### 2.3 Histological, immunohistochemistry analysis and senescence-associated staining

Rectal tissues were collected, formalin-fixed, and paraffin-embedded for histological evaluation. Hematoxylin and eosin (H&E) and Masson’s Trichrome staining were used to assess radiation-induced mucosal injury, inflammatory infiltration, and fibrosis. Specific features, including Goblet cell depletion, mucosal erosion, transmural inflammation, and reactive atypia, were evaluated by a pathologist. Masson’s Trichrome–stained sections were scanned using a ZEISS Axioscan 7 microscope (Carl Zeiss Microscopy GmbH, Germany) equipped with Zen 2 software. Images were processed with ImageJ software (National Institutes of Health, USA). Fibrosis was quantified by calculating the ratio of blue pixels, representing collagen deposition, relative to the total tissue area.

Immunohistochemistry was performed to assess macrophage polarization, using antibodies against inducible nitric oxide synthase (iNOS, M1 macrophages) and CD163 (M2 macrophages). Formalin-fixed, paraffin-embedded tissue sections were deparaffinized and rehydrated, followed by heat-induced antigen retrieval in citrate buffer (pH 6.0). Endogenous peroxidase activity was blocked using 3% hydrogen peroxide for 10 minutes. Non-specific binding was minimized with 5% bovine serum albumin prior to overnight incubation at 4 °C with primary antibodies against iNOS (1:200 dilution, Abcam) or CD163 (1:200, Abcam). After washing, HRP-conjugated secondary antibodies were applied, and signal detection was performed using DAB substrate. Sections were counterstained with hematoxylin and examined under a light microscope. Macrophage infiltration was quantified by counting positively stained cells in five randomly selected high-power fields per sample.

### 2.4 Culture and polarization of bone marrow–derived macrophages

Bone marrow cells were harvested from 8-week-old BALB/c mice (source/reference) and cultured in Dulbecco’s Modified Eagle Medium supplemented with 10% fetal bovine serum and 25 ng/mL macrophage colony-stimulating factor (CSF-1). After six days of culture, interleukin-4 was added at a final concentration of 25 ng/mL for 24 hours to promote M2 macrophage polarization from M0 macrophages. To induce cellular senescence, differentiated BMDMs were exposed to a single 15 Gy dose of irradiation. Cells were subsequently incubated for 72 hours prior to senescence staining.

### 2.5 Assessment of radiation-induced senescence in BMDMs

To assess the effects of MeBu, metformin and butyrate on radiation-induced senescence, BMDMs were isolated from murine femurs and cultured under standard conditions. Cells were exposed to ionizing radiation and subsequently treated with 4 mM MeBu, 4 mM metformin or 4 mM butyrate for 48 hours. Total RNA was extracted from treated and control cells, and cDNA was synthesized for quantitative real-time PCR (qPCR) analysis. The expression of senescence-associated genes, including *p16* and *p21*, was quantified using SYBR Green–based detection. Gene expression levels were normalized to housekeeping genes and analyzed to determine treatment effects on radiation-induced senescence.

### 2.6 Senescence-associated β-galactosidase staining

Cellular senescence of senescence-associated β-galactosidase (SA-β-Gal) was assessed using the Senescence Detection Kit (ab65351, Abcam, UK) following the manufacturer’s instructions. The X-Gal staining solution was freshly prepared and added to each well (400 μL per well in 24-well plates). To minimize evaporation, plates were sealed with parafilm and incubated in a dry 37 °C incubator without CO_2_ for 8 hours. After incubation, cells were examined under a bright-field microscope. Senescent cells were identified by characteristic blue cytoplasmic staining, indicating SA-β-Gal activity at pH 6.0.

### 2.7 Quantitative real-time PCR

Total RNA was extracted using an RNA purification kit (RNA MicroPrep w/TriReagent, ZYMO RESEARCH,US), and reverse transcription was performed using Moloney Murine Leukemia Virus Reverse Transcriptase (M-MLV RT, Invitrogen, Carlsbad, CA, USA) at 37 °C for 50 minutes. Quantitative PCR was carried out using a SYBR Green PCR Master Mix (FastStart Universal SYBR Green Master (Rox), Roche, Switzerland) on a real-time PCR system. Primer sequences were as follows:

GAPDH: forward 5′-AGGTCGGTGTGAACGGATTTG-3′, reverse 5′-TGTAGACCATGTAGTTGAGGTCA-3′.

p16: forward 5’-CTGGGTGCTCTTTGTGTT, reverse 5’-GTGCTTGAGCTGAAGCTATG.

p21: forward 5’-GTGGGTGTCAAAGCACTTAG, reverse 5’-ACAGTCCAGACCAGGATGTTA.

Relative gene expression was calculated using the ΔCt method, normalizing the target gene Ct values to GAPDH. All samples were analyzed in triplicate.

### 2.8 Integrated senescence burden index analysis

To integrate transcriptional senescence markers into a composite metric, a normalized SBI was established based on the expression of p16 and p21. Quantitative real time PCR data were normalized to internal controls and subsequently to the irradiated group (defined as 1.0). The SBI for each individual sample was calculated as the arithmetic mean: SBI = (Normalized p16 + Normalized p21)/2. Equal weighting was assigned to *p16* and *p21* to ensure a balanced representation of the *p16/Rb* and *p53/p21* axis, the two primary molecular pathways that independently mediate permanent cell cycle arrest (23). This exploratory metric was validated by its alignment with the visual trends of SA-β-Gal staining, providing a quantifiable molecular surrogate that complements qualitative histological observations.

### 2.9 Data and statistical analysis

Data are expressed as the mean ± standard error of the mean (SEM), except for histopathological and colonoscopy scores, which are reported as median (range). The sample size for each experimental group was n = 9. After confirming data normality, multiple group comparisons were performed using one-way analysis of variance (ANOVA) followed by Tukey’s *post hoc* test. For comparisons between two groups, Student’s t-test or the Mann-Whitney U-test was used as appropriate. The Mantel-Cox log-rank test was used to assess differences between survival curves. Statistical significance was defined when the p-value was < 0.05. All statistical analyses were performed using GraphPad Prism version 8 (San Diego, CA, United States).

## 3 Results

### 3.1 Characterization of the chronic RP model by 15 Gy brachytherapy

**Figure 1:**
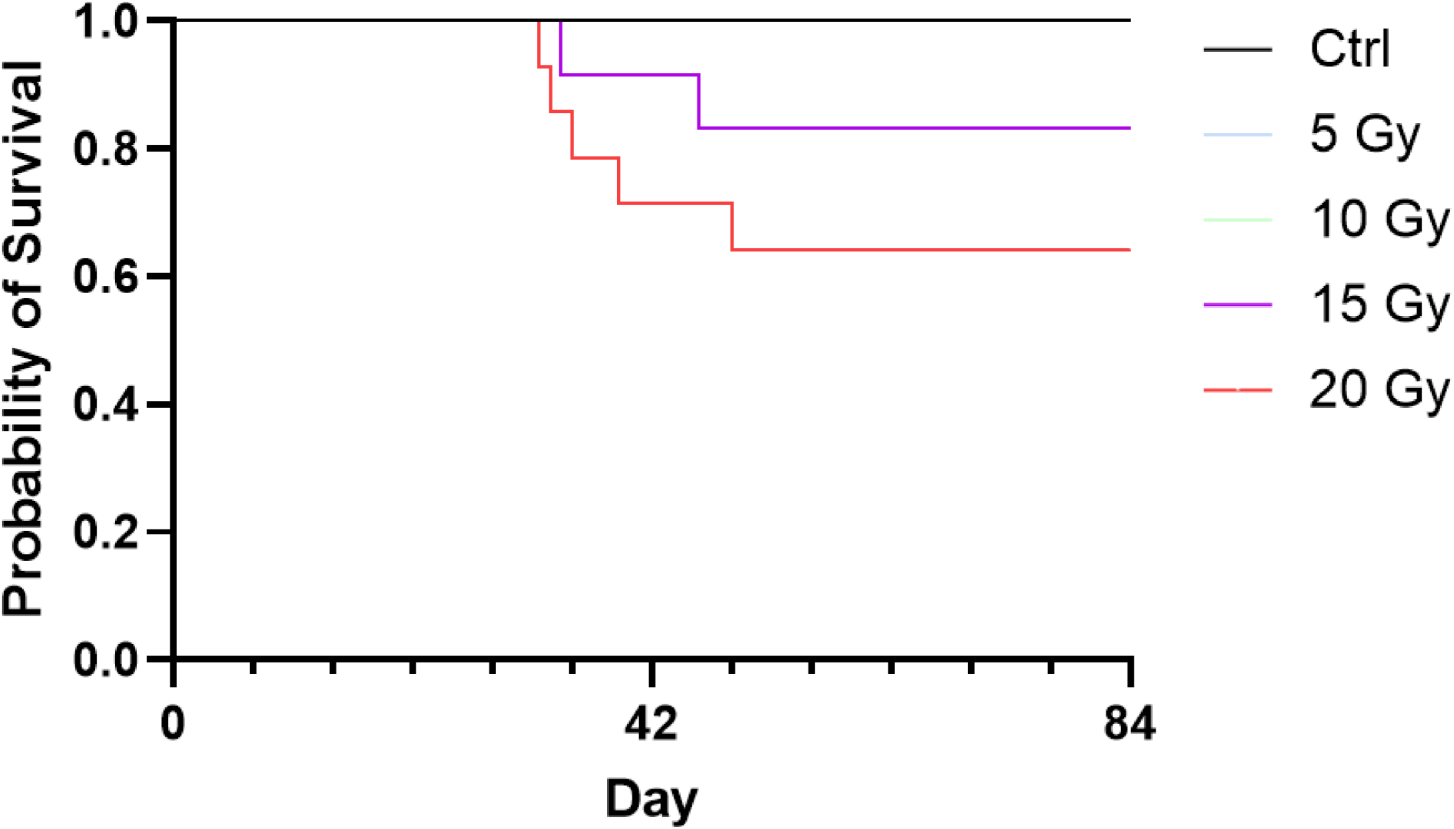
Survival outcomes following HDR brachytherapy at varying radiation doses. Mice received a single fraction of 0 (control), 5, 10, 15, or 20 Gy using high-dose-rate (HDR) brachytherapy (n = 10, 10, 16, and 14, respectively). By 12 weeks post-irradiation, survival remained 100% in the control, 5 Gy, and 10 Gy groups. Survival rates declined to 87.5% in the 15 Gy group and 64.3% in the 20 Gy group.

To establish a reproducible murine model of chronic RP, we first evaluated the impact of varying radiation doses (5, 10, 15, and 20 Gy) with numbers of 10, 10, 16, and 14 in each group, respectively. A dose-dependent increase in morbidity and mortality was observed; by 12 weeks, survival was 100% in the 5 and 10 Gy groups, but decreased to 87.5% (14/16) and 64.3% (9/14) in the 15 and 20 Gy groups (**Figure 1**). While 20 Gy induced excessive transmural necrosis and structural disruption, 15 Gy successfully produced hallmark features of chronic RP with an overall survival rate of 82% (37/45), making it the optimal dose for balancing clinical relevance and experimental viability.

**Figure 2:**
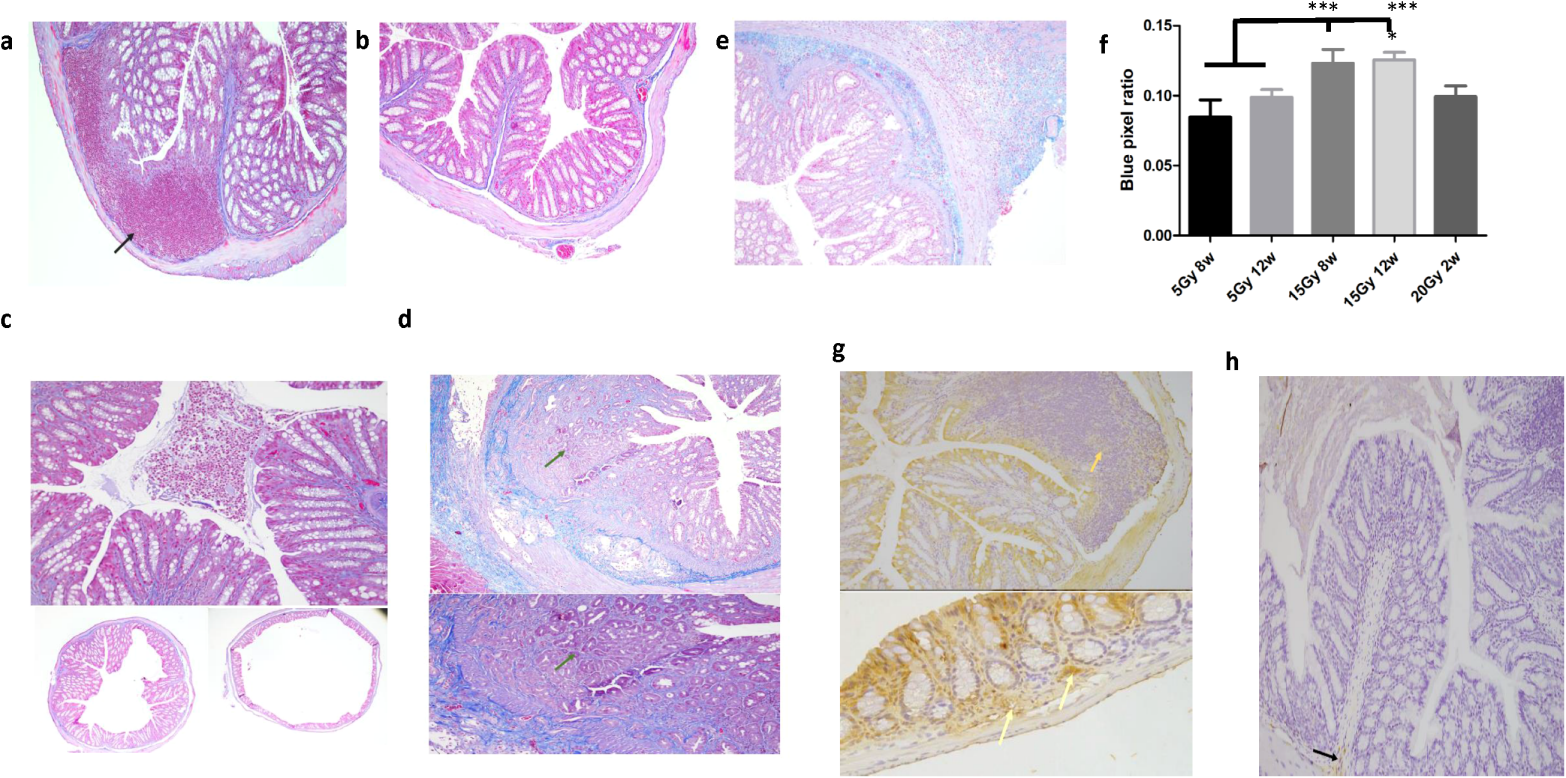
Histopathological changes and macrophage polarization in rectal tissue following HDR brachytherapy. **(a)** 5 Gy at 8 weeks: Acute inflammatory response with neutrophil infiltration and focal hemorrhage (black arrow). **(b)** 5 Gy at 12 weeks: Resolution of acute inflammation with reduced vascular congestion and minimal residual hemorrhage. **(c)** 15 Gy at 8 weeks: Extensive mucosal erosion with marked transmural inflammatory infiltration. **(d)** 15 Gy at 12 weeks: Chronic injury characterized by goblet cell depletion and reactive epithelial atypia (green arrow). **(e)** 20 Gy at 2 weeks: Severe transmural inflammation with prominent submucosal edema. **(f)** Quantitative analysis of the blue pixel ratio from Masson’s Trichrome staining showed significantly increased fibrosis in the 15 Gy group at both 8 and 12 weeks (p = 0.0443, p = 0.0097). **(g)** iNOS staining demonstrates marked infiltration of pro inflammatory M1 macrophages (gold arrows). **(h)** CD163 staining shows reduced presence of anti-inflammatory M2 macrophages (black arrows). Data are presented as mean ± SEM (n = 9 per group). ***: statistically significant.

Histopathological analysis further characterized the progression of tissue injury at this dose. In the 15 Gy group, we observed persistent mucosal erosion, transmural inflammation, and goblet cell depletion that progressed from 8 to 12 weeks post-irradiation. This was accompanied by significant fibrotic remodeling, as confirmed by an increased blue pixel ratio in Masson’s Trichrome staining (p = 0.0443 at 8 weeks; p = 0.0097 at 12 weeks; **Figures 2a–f**). Furthermore, immunohistochemistry revealed that 15 Gy irradiation shifted the immune microenvironment toward a pro-inflammatory state, characterized by an abundance of iNOS-positive M1 macrophages and a reduction in CD163-positive M2 macrophages (**Figures 2g, h**). Together, these findings demonstrate that 15 Gy HDR brachytherapy effectively establishes a chronic RP model driven by sustained inflammation and macrophage dysregulation.

### 3.2 MeBu enema ameliorates radiation-induced inflammation and fibrosis

**Figure 3:**
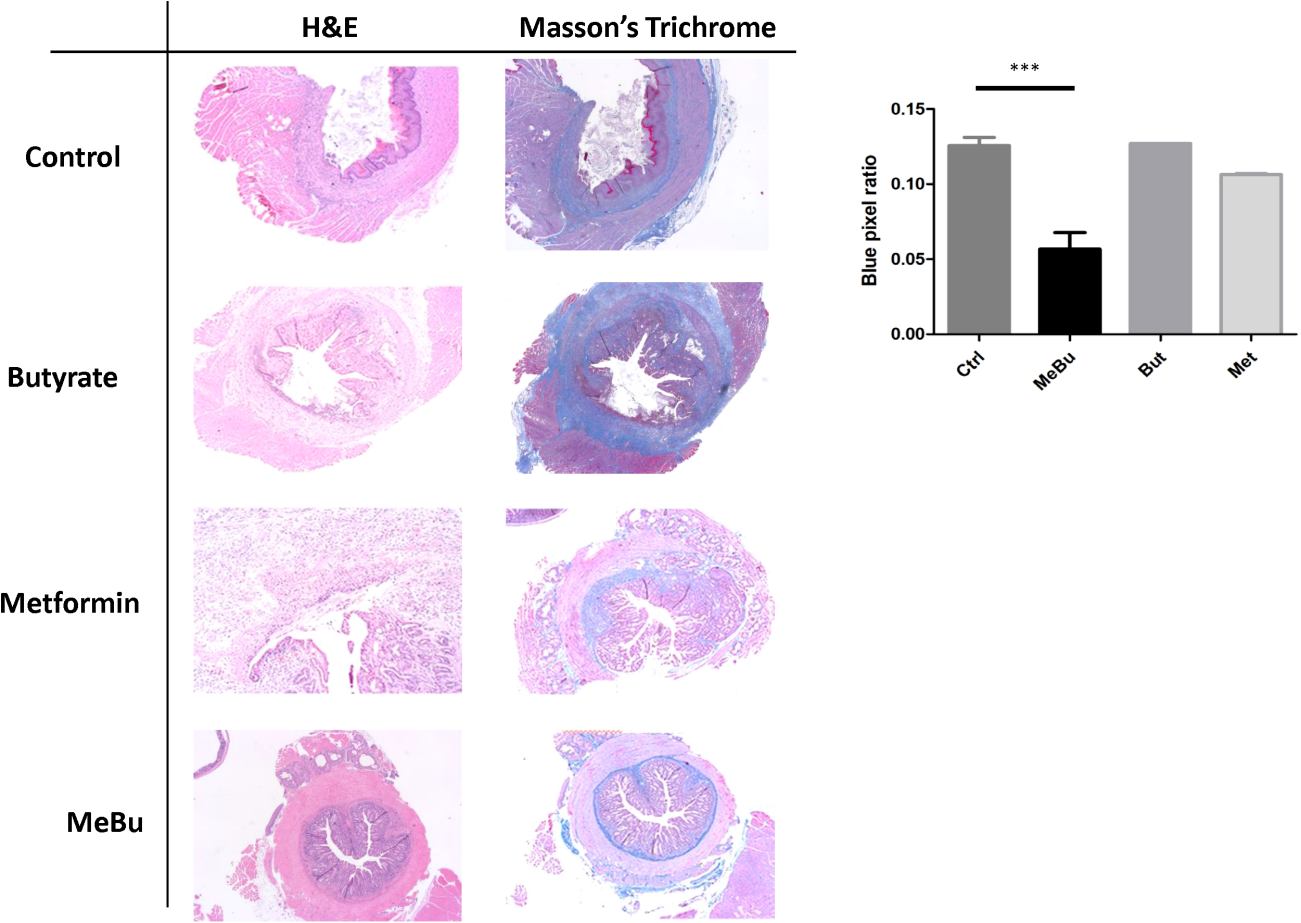
Histological evaluation of therapeutic responses to rectal enemas following 15 Gy irradiation. MeBu treatment (weeks 4–8) markedly reduced fibrosis, restored mucosal integrity, and minimized inflammation compared with control (15Gy), metformin or butyrate group. Blue pixel analysis confirmed a significant reduction in fibrosis (p = 0.0027). Data are presented as mean ± SEM (n = 9 per group). ***: statistically significant.

**Figure 4:**
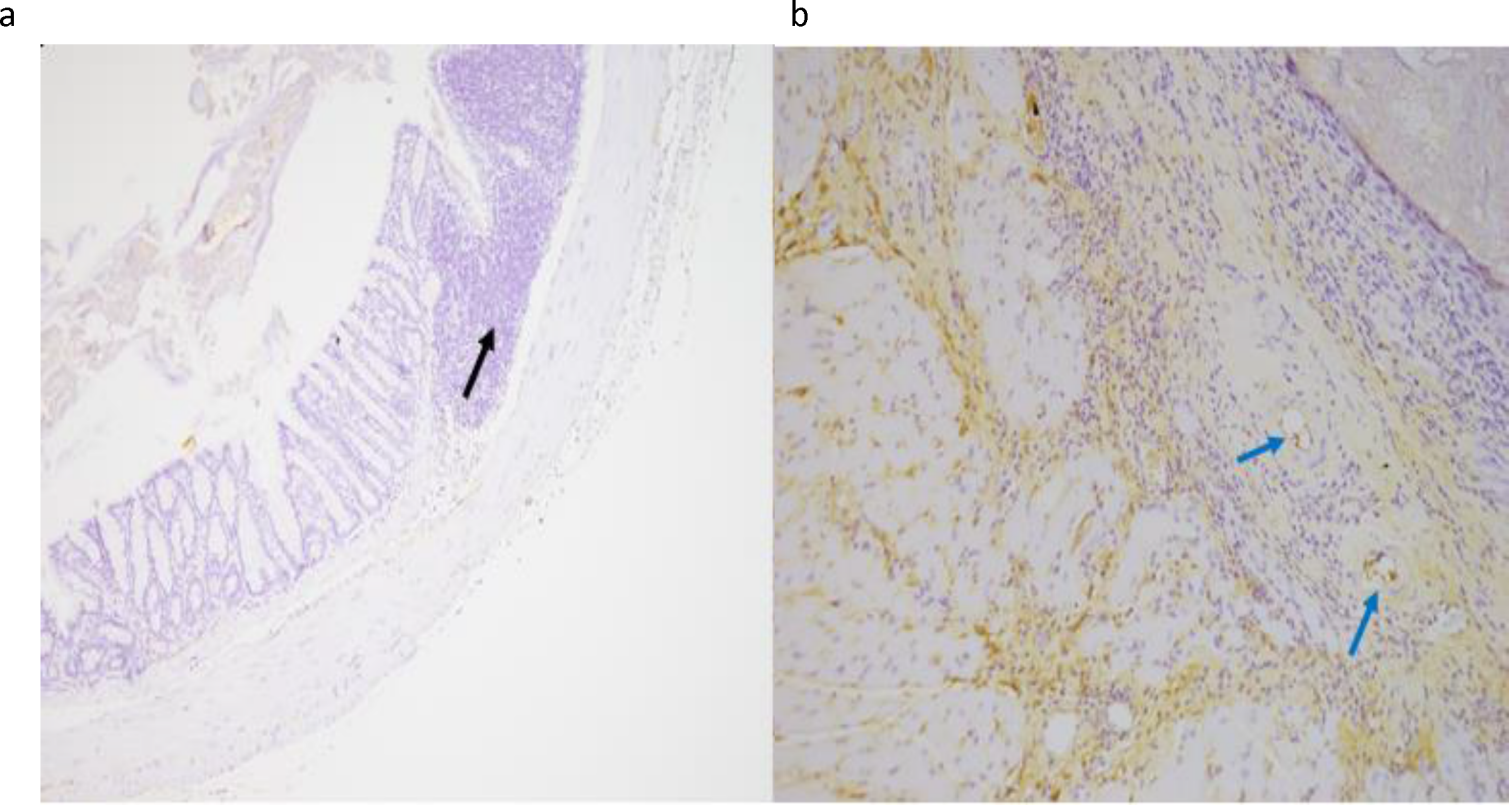
MeBu shifts macrophage polarization toward an anti-inflammatory profile. **(a)** iNOS staining of rectal tissue demonstrated decreased M1 macrophage infiltration (black arrows) in the MeBu group. **(b)** CD163 staining showed increased M2 macrophage (blue arrows) in the MeBu group. Representative images are shown from n = 9 mice per group.

To assess therapeutic efficacy, mice received daily rectal enemas of metformin, butyrate, or MeBu from weeks 4 to 8 following 15 Gy irradiation. Macroscopic examination at week 12 revealed that MeBu treatment significantly mitigated radiation-induced perianal swelling and rectal congestion, while also restoring normal fecal consistency compared to the severe diarrhea observed in the irradiated control group (**Supplementary Figures 1a, b**). Histological evaluation with H&E and Masson’s Trichrome staining demonstrated modest improvements in tissue integrity and collagen deposition in the metformin- and butyrate-treated groups. In contrast, MeBu treatment produced the most significant therapeutic benefit, with markedly reduced fibrosis, restoration of mucosal architecture, and minimal residual inflammation or ulceration. High-magnification H&E imaging further confirmed the preservation of well-organized crypt structures in the MeBu group, which were nearly absent in the 15 Gy radiation group (**Supplementary Figure 1c**). Quantitative analysis of the blue pixel ratio confirmed a statistically significant reduction in fibrosis in the MeBu group (*p* = 0.0027; **Figure 3**). Immunohistochemical staining further revealed reduced M1 macrophage infiltration (iNOS-positive) and an increased population of M2 macrophages (CD163-positive) following MeBu treatment (**Figure 4**), suggesting a phenotypic shift toward an anti-inflammatory and tissue-reparative profile. Since chronic M1-driven inflammation is often sustained by the SASP, we next investigated whether these effects were mediated through the modulation of cellular senescence.

### 3.3 MeBu reduces senescence markers in irradiated BMDMs

**Figure 5:**
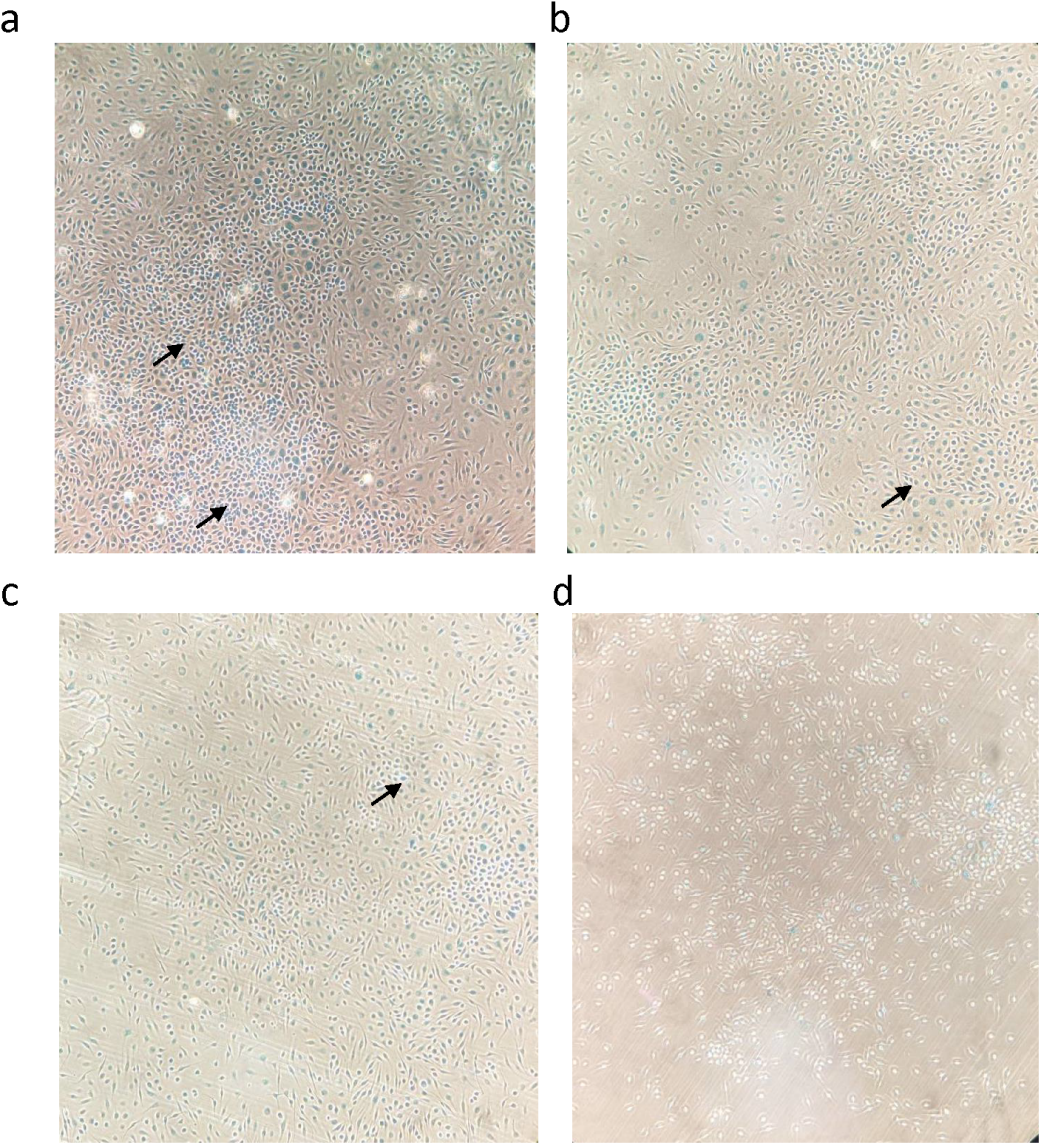
MeBu reduces SA-β-Gal–positive cells expression in irradiated BMDM. Representative SA-β-Gal staining images of BMDMs 72 hours after 15 Gy irradiation **(a)** and treatment with metformin **(b)**, butyrate **(c)**, or MeBu **(d)**. Prominent blue staining indicates senescent cells (black arrows). MeBu group showed markedly decreased SA-β-Gal–positive cells. Experiments were performed in three independent replicates (n = 9 total per condition).

**Figure 6:**
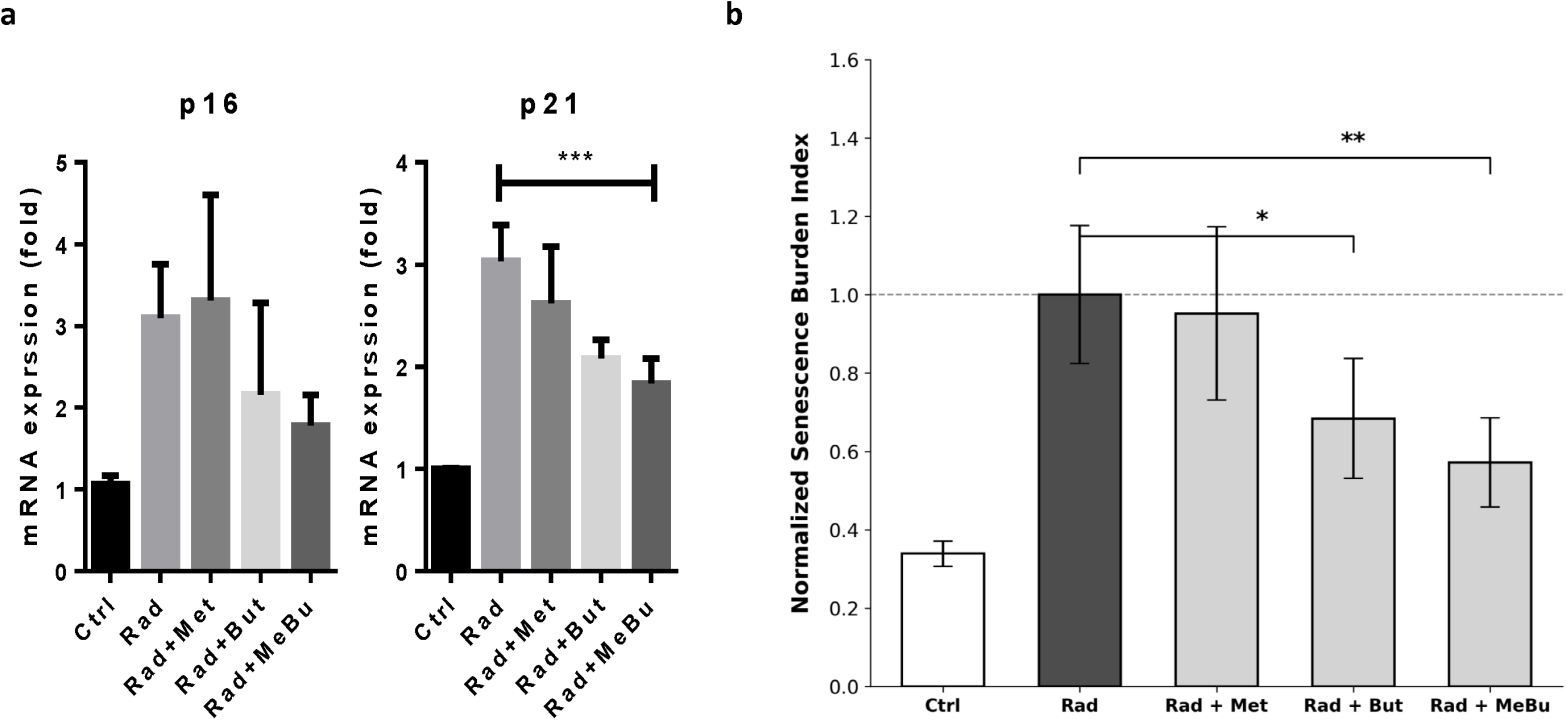
Molecular assessment of cellular senescence and the integrated senescence burden index (SBI). **(a)** Radiation significantly upregulated both *p16* and *p21* expression. MeBu treatment significantly reduced *p21* expression (*p* = 0.0074), while *p16* expression showed a non-significant downward trend (*p* = 0.0568) and approached levels observed in non-irradiated controls. ***: statistically significant. **(b)** The SBI calculated as the arithmetic mean of normalized *p16* and *p21* levels, providing a composite metric of the overall senescence burden across treatment groups (Rad group = 1.0). While metformin (Rad + Met) showed minimal effect, butyrate (Rad + But) and especially the Rad + MeBu combination significantly attenuated the senescence burden. Data are presented as mean ± SEM (n = 9 per group).* p < 0.05, ** p < 0.01.

To investigate these mechanisms in a controlled setting, BMDMs were used to simulate the infiltrating monocytes recruited to the irradiated rectum. This model allows for reliable quantification of senescence markers, which is technically challenging using primary intestinal macrophages due to low cell yields. BMDMs were irradiated with 15 Gy and subsequently treated with metformin, butyrate, or the MeBu combination. Senescence-associated β-galactosidase (SA-β-Gal) staining revealed robust blue precipitates in irradiated BMDMs, indicating strong induction of senescence. Treatment with metformin or butyrate alone produced only modest reductions in staining intensity, whereas MeBu co-treatment nearly abolished SA-β-Gal positivity, suggesting a marked attenuation of radiation-induced senescence (**Figure 5**). Quantitative PCR showed that both p16 and p21 mRNA were significantly upregulated in irradiated BMDMs compared with non-irradiated controls. MeBu treatment significantly suppressed p21 expression (p = 0.0074), restoring levels close to baseline. Although p16 expression did not reach statistical significance compared with irradiated controls (p = 0.0568), MeBu treatment produced a downward trend, with expression levels comparable to those in non-irradiated cells (**Figure 6a**). Together, these results demonstrate that MeBu exerts a stronger senescence-modulating effect than either agent alone, effectively mitigating radiation-induced senescence in macrophages.

### 3.4 Integrated SBI reveals attenuation of radiation-induced macrophage senescence by MeBu

To further integrate the effects of MeBu on radiation-induced senescence, a composite SBI was calculated using *p16* and *p21* expression levels. As an exploratory metric, the SBI reflects the collective activation of the two primary senescence pathways. As shown in **Figure 6b**, irradiation markedly increased the senescence burden in BMDMs compared with non-irradiated controls. While treatment with metformin alone showed limited impact, butyrate treatment significantly reduced the SBI (p < 0.05 vs. Radiation group). Notably, the MeBu combination produced the most robust attenuation of the senescence burden (p < 0.01 vs. Radiation group), with index values approaching those of non-irradiated controls. These results show that MeBu synergistically suppresses senescence. The SBI data correlate with the SA-β-gal staining in **Figure 6a**, together confirming the combination’s potent anti-senescent efficacy.

## 4 Discussion

This study is the first to demonstrate the therapeutic efficacy of MeBu in chronic RP, advancing beyond traditional anti-inflammatory approaches by showcasing its synergistic effects on macrophage phenotypic shifts and senescence modulation.

The chronic RP model developed using a single 15 Gy fraction of HDR brachytherapy induced significant inflammatory and fibrotic changes. This approach offers several advantages: it minimizes applicator positioning errors, avoids mechanical injury associated with repeated fractionation, and does not require specialized external radiotherapy equipment (24, 25). The 2 mm diameter applicator reduces the risk of overdilation-related injury. Biologically, the 15 Gy single dose is equivalent to approximately 54 Gy conventional fractionation (assuming an α/β ratio of 3), a dose within the tolerable range for small-volume rectal irradiation (26). The 18% mortality rate at 12 weeks is significantly lower than the 50% to 86% reported in other fractionated protocols (25, 27). Another study using the X-RAD 225Cx system reported similar inflammatory outcomes, but their reliance on specialized irradiators limits reproducibility compared to our accessible applicator-based method (24).

The synergistic effects of MeBu may be significant by four primary mechanisms: anti-inflammatory action, senescence modulation, tight junction restoration, and epigenetic regulations. Regarding anti-inflammatory effects, our observation of an elevated M2 macrophage proportion aligns with evidence that metformin promotes M2 polarization via the AMPK/mTOR/NLRP3 signaling axis (15, 16, 28, 29). Butyrate facilitates wound repair through G-protein receptor activation and AMPK signaling (30–32), modulates M2 macrophage polarization and enhancing regulatory T-cell functions while inhibiting excessive neutrophil infiltration (33). These findings provide a mechanistic basis for the clinical efficacy of MeBu observed in chronic RP patient unresponsive to conventional steroid enema (20).

The senescence modulating effects of MeBu represent a pivotal mechanistic component of its therapeutic profile. While transient senescence initially constrains tissue injury, persistent accumulation of senescent macrophages sustains chronic inflammatory signaling and impairs tissue resolution (34, 35). Our data show that 15 Gy radiation induced a robust senescent phenotype, characterized by increased SA-β-Gal and *p16/p21* expression, which MeBu effectively attenuates via a senomorphic strategy. By utilizing the SBI, we show that the MeBu combination more effectively recalibrates the pro-senescent environment than either metformin or butyrate alone. Beyond its role in experimental quantification, the SBI holds significant potential as a translational tool (23, 36), by integrating multiple biomarkers, could help identify patients at high risk for RAVE and facilitate the efficacy evaluation of anti-senescent therapies in clinical settings.

Ionizing radiation promotes monocyte recruitment and M1 infiltration, exacerbating senescent cell accumulation and impairing endothelial integrity (37–39). The MeBu combination exhibits superior efficacy by modulating the *p*16 and *p*21 axes central to radiation-induced tissue aging (40, 41). Regarding clinical safety, both agents possess documented anti-tumor properties. Metformin inhibits malignant growth via AMPK activation (42), while butyrate acts as an HDAC inhibitor to induce cancer cell apoptosis (43). These dual mechanisms suggest that MeBu treatment is unlikely to compromise oncological control and may offer synergistic anti-cancer benefits (19).

Finally, we hypothesize that MeBu stabilizes the mucosal microenvironment through tight junction restoration and epigenetic modulation. In chronic RP, barrier dysfunction leads to increased permeability and vascular ectasia (44). Metformin and butyrate both accelerate tight junction reassembly via AMPK-dependent pathways (18, 45, 46). As an HDAC inhibitor, butyrate may reprogram the epigenetic state toward a reparative macrophage phenotype (47, 48), potentially counteracting the reactivation of fetal-like transcriptional programs in chronic intestinal injury (47, 48). While these pathways offer a compelling explanation for the senomorphic effects, they remain hypothesis-generating and require further mechanistic validation. Several limitations should be acknowledged. First, while iNOS and CD163 provided a baseline phenotype, they do not capture the full functional heterogeneity of macrophages; future studies using markers like Arg1, TREM2, or MerTK would provide a more granular view of the anti-fibrotic transition. Second, although senescence was evidenced by SA-β-Gal, *p16*, and *p21*, we did not directly profile SASP factors (e.g., IL-6, TNF-α) or DNA damage markers (e.g., γH2AX). Additionally, the use of 8 week old mice was intended to ensure a standardized injury model by minimizing biological variability; however, this may not fully reflect the ‘inflammaging’ baseline seen in elderly patients. Further validation in aged models or clinical cohorts is therefore warranted.

## 5 Conclusion

Our findings suggest that combined MeBu approach may attenuate radiation-induced senescence-associated macrophage dysfunction in a murine chronic RP model. The therapeutic benefit of MeBu was characterized by a reduction in mucosal fibrosis and a phenotypic shift toward reparative M2-like macrophages. The significant decrease in the integrated SBI indicates that MeBu could effectively modulate *p16* and *p21* in irradiated macrophages. While these results are promising, further translational investigations are necessary to confirm the clinical potential of this metabolic intervention for chronic RP.

## Data Availability

The raw data supporting the conclusions of this article will be made available by the authors, without undue reservation.
