# Supplementary material for: Metformin and butyrate attenuate chronic radiation proctitis by alleviating inflammation and macrophage senescence
Source: Front Immunol. 2026 Apr 2;17:1802803. doi: 10.3389/fimmu.2026.1802803 (PMC13083195; doi:10.3389/fimmu.2026.1802803)
Supplement: Supplementary file 1 [file DataSheet1.pdf]

a

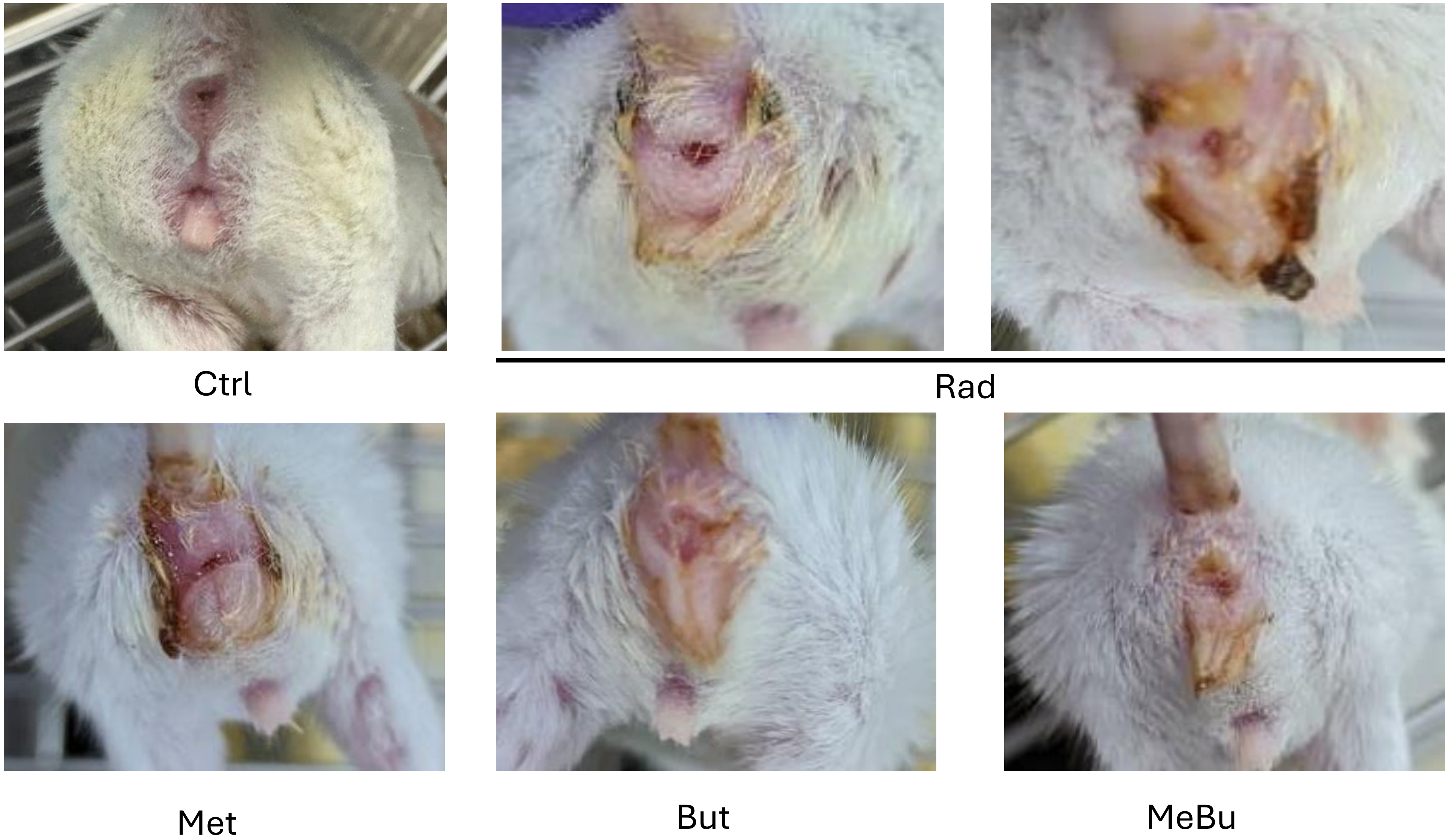

b

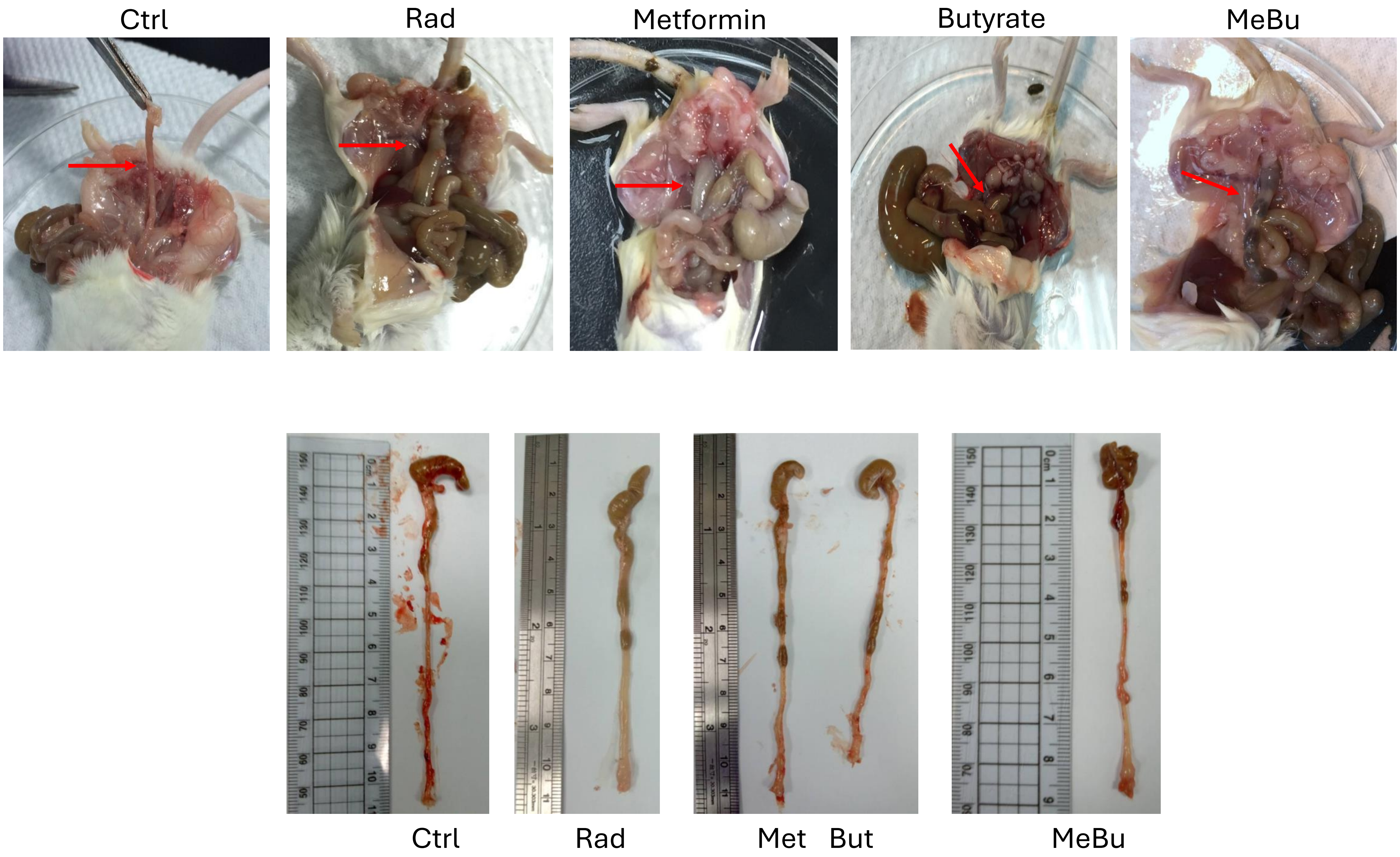

c

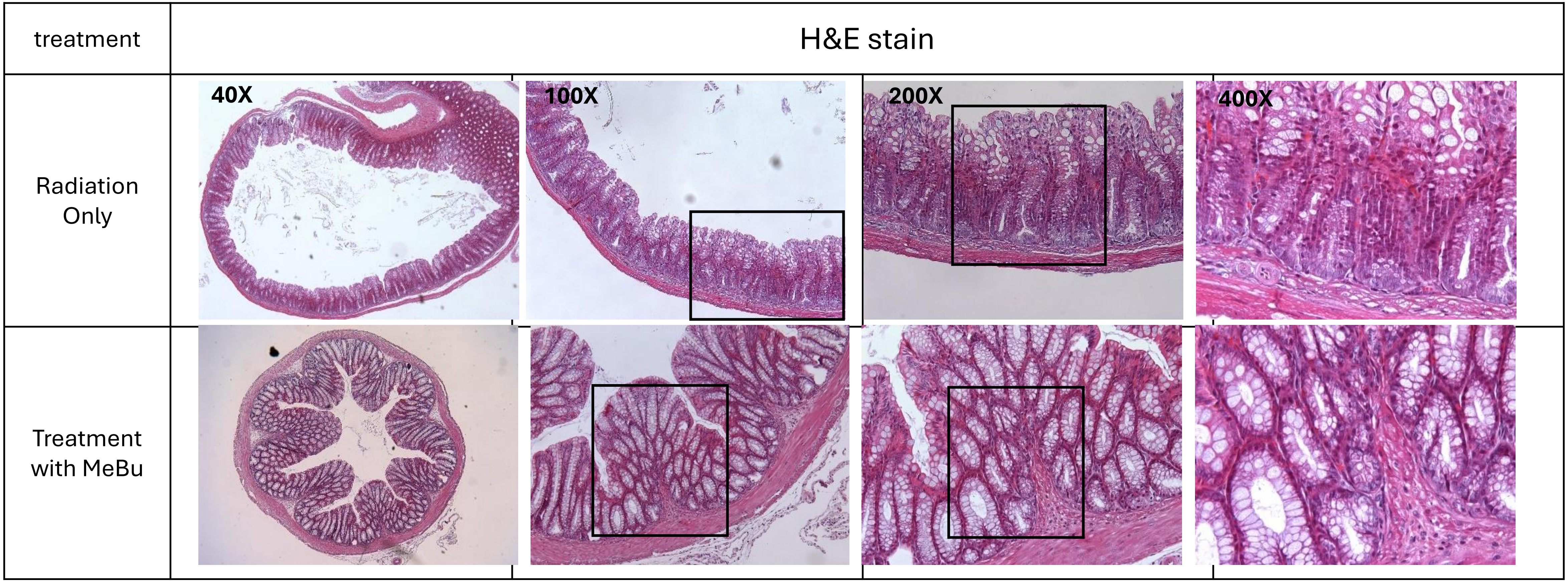
treatment
H&E stain
Radiation Only
Treatment with MeBu
40X
100X
200X
400X
